# Institutionalized elderly are able to detect small viscosity variations in thickened water with gum-based thickeners: should texture classifications be reviewed?

**DOI:** 10.1186/s12877-021-02599-8

**Published:** 2021-11-19

**Authors:** Fernando Calmarza-Chueca, Ana Cristina-Sánchez-Gimeno, Javier Perez-Nogueras, Alberto Caverni-Muñoz, Alejandro Sanz-Arque, José Miguel Arbones-Mainar, Alejandro Sanz-Paris

**Affiliations:** 1 Department of Endocrinology and Nutrition, Miguel Servet Hospital, Zaragoza, Spain; 2 Food Technology, Faculty of Veterinary, Zaragoza University, AgriFood Institute of Aragon (IA2), C/ Miguel Servet, 177, 50013 Zaragoza, Spain; 3 Geriatric Unit, Elias Martinez Nursing Home, Zaragoza, Spain; 4 Diet Service, Renal Patients Association, Alcer Ebro, Zaragoza, Spain; 5 Aragón Health Research Institute/ Instituto de Investigación Sanitaria (IIS) Aragón, Zaragoza, Spain; 6 Adipocyte and Fat Biology Laboratory (AdipoFat), Translational Research Unit, University Hospital Miguel Servet, Instituto Aragones de Ciencias de la Salud (IACS), 50009 Zaragoza, Spain; 7 Center for Biomedical Research in Network Physiopathology Obesity and Nutrition (CIBERObn), Institute of Health Carlos III (ISCIII), Madrid, Spain

**Keywords:** Deglutition disorders, Dysphagia, Institutionalized elderly population, Rheology, Sensory analysis, Gum-based thickener, Viscosity

## Abstract

**Background:**

The prevalence of dysphagia is very high in institutionalized elderly. Knowledge of the rheological and sensory characteristics of the various thickeners in elderly is limited, although it has been seen that there are differences between the rheological behaviors of gum-based thickeners with different composition. Moreover, we have not found sensory studies of viscosity in institutionalized elderly. Our hypothesis was that viscosity ranges established by the scientific societies, such as the National Dysphagia Diet Task Force (NDD), seem to be very wide and individuals might be able to detect small differences within the same texture range. The objectives of our study were 1) comparing the rheological characteristics of two commercial gum-based thickeners with different composition, dissolved in water under standard conditions, and 2) perform a sensory analysis (with both adults and institutionalized elderly) to detect different viscosities within the same texture (nectar and honey).

**Methods:**

Two commercial thickeners based on gums (NC and RC) were studied analyzing their viscosity in water with different concentrations (shear rate: 50 s^− 1^; temperature: 22–25 °C). A sensory analysis involving 26 elderly and 29 adult controls was carried out to evaluate whether differences within nectar and honey textures among gum-based thickeners could be distinguished.

**Results:**

As the shear rate increases, viscosity decreases (non-Newtonian and pseudoplastic behavior). At the same concentration, each thickener produces a different viscosity (*p* < 0.05). Institutionalized elderly detected viscosity differences in nectar range of 49.9 (2.5) mPa·s (*p* < 0.05) and 102.2 (4.7) mPa·s (*p* < 0.0001). They also detected viscosity differences in honey texture range of 134.6 (9.7) mPa·s (*p* < 0.05) y 199.3 (9.2) mPa·s (*p* < 0.0001). Their caregivers also detected viscosity differences in both viscosity ranges (*p* < 0.0001) and with greater intensity than the elderly in honey texture (p: 0.016).

**Conclusions:**

Our results suggest that the accepted viscosity ranges by NDD for the different textures might be too wide because institutionalized elderly and their caregivers are able to discern small differences in viscosity in nectar and honey textures. Gum-based thickeners with different composition showed differences in viscosity capacity, so they are not interchangeable.

## Clinical relevance statement

In this work we observed that at the same concentration, each gum-based thickener produces a different viscosity that even elderly people are capable of discriminating. This suggests that the viscosity ranges accepted by National Dysphagia Diet Task for different textures could be too wide. The viscosity of two gum-based thickeners commonly used in clinical practice with water as a solvent varies, depending on their composition and concentration. These facts can be used to optimize the prescription of each thickener. Thickeners are not interchangeable even if they belong to the same group (gum-based thickeners).

## Background

Dysphagia is a very frequent clinical symptom in the elderly population and in patients with pathologies such as neurodegenerative diseases, dementia, stroke or some types of cancer. It is estimated that it affects between 15 and 70% in institutionalized elderly [1]. There is a variability of the percentage depending on the country where this population is found since there are differences in lifestyle [2]. From the pathophysiological point of view, dysphagia occurs mainly because of obstructive lesions or motor disorders [3]. Dysphagia can have very important clinical consequences including aspiration pneumonia, malnutrition, dehydration, and even psychological problems. In many patients with dysphagia, thickening powders are added to the liquids they ingest in order to increase their consistency and viscosity, decrease the flow rate of the bolus during swallowing and prevent its passage into the airway [4]. However, the optimal viscosity of the bolus, which ensures optimal swallowing, has not been optimally established according to the type of dysphagia or its severity [4].

On the other hand, rheology is a discipline that studies the deformation of materials in response to external forces, and allows for the determination of material properties such as elasticity and viscosity. In the field of nutrition, it is important to determine the rheological characteristics of the alimentary bolus because they condition to a great extent the swallowing process [4]. There is clinical evidence suggesting that an increase in the viscosity of the alimentary bolus reduces the risk of aspiration [4]. In this sense, the National Dysphagia Diet Task Force (NDD) [5] defined four levels of thickened liquids that are frequently used in clinical practice. The classification and ranges are based on shear viscosities measured at one single shear rate of 50 s^− 1^ and at a temperature of 25 °C. In this sense, they proposed four consistency levels: 1) Thin for viscosities lower than 50 mPa·s; 2) Nectar-like for viscosities in the range of 51–350 mPa·s; 3) Honey-like for viscosities in the range of 351–1750 mPa·s; and 4) Spoon-thick or pudding for viscosities above 1750 mPa·s. Subsequently, a syringe flow test was developed by the International Dysphagia Diet Standardization Initiative (IDDSI). This new test classifies thickened fluids into five categories: thin, slightly thick, mildly thick, moderately thick, and extremely thick. Its aim is to create a new global standardized terminology, easy to use by end users such as caregivers, patients, and clinicians without a rheological delineation [6].

For the various commercial thickeners distinct concentrations of product are recommended to reach the nectar, honey or pudding texture. However, as their composition is different, their rheological properties might also differ. In this way, a liquid thickened with two commercial thickeners in nectar texture, for example, could have a different degree of viscosity measured by rheology, and this difference could be clinically significant.

Comparative data on the rheological properties of the different commercial gum-based thickeners are not scarce [7, 8]. Currently, there are some works comparing the rheological results with the sensory results in thickeners [9–11] but none involving institutionalized elderly where dysphagia is very prevalent. Our hypothesis was that viscosity ranges established by NDD seem to be very wide and individuals, both old and young, might be able to detect small differences within the same texture range.

The aims of this work were: 1) Compare the rheological characteristics of two commercial gum-based thickeners with different composition, dissolved in water and under standard conditions; 2) Test with the use of sensory analysis (both with adults and elderly) whether it is possible to detect different viscosities within the same texture (nectar and honey).

## Methods and analysis

### Thickeners

Two types of commercial thickeners were used, whose composition is shown in Table 1. We used two third generation thickeners (NC and RC) with different types of gums and without starch. The main ingredients of each one of the thickeners are the followings: 1) NC: maltodextrins, guar gum, xanthan gum, potassium chloride and sodium chloride; 2) RC: maltodextrins, xanthan gum, sodium chloride and potassium chloride.

**Table 1:** Commercial name and nutritional values of thickeners used in the study

Composition per 100 g of the product	Thickener NC	Thickener RC
Commercial name	Nutilis clear®	Resource thickener clear®
Kcal	290 kcal	306 kcal
Protein	0.8 g	1 g
Fat	0 g	0 g
Carbohydrates	57.6 g	62 g
Sugar	3.7 g	1.8 g
Fiber	28 g	27 g
Sal	3.8 g	2.7 g
Na	1500 mg	1060 mg
K	< 40 mg	400 mg

### Solvent

The thickeners were dissolved in commercial mineral water from the Sacalm spring in Sant Hilari (Girona, Spain). This water is sold under the commercial name of Fontvella® and has the following composition: calcium 43.2 mg/l, magnesium 11.5 mg/l, sodium 12.3 mg/l, bicarbonates 167 mg/l. It also has a conductivity of 303 μS/m.

### Preparation of the samples

Water and thickeners were mixed in a shaker specially made for use in this study, 16 cm high, 22 cm in diameter and with 400 ml capacity. A Nahita Blue Series 5173 electronic precision weighing scale was used to weigh the samples. The technique for the preparation of the samples consisted of agitating the shaker 15 times with an approximate arch of 50 cm, intending to reproduce the real conditions of preparation as accurately as possible.

The concentrations used for the preparation of the samples in water were between a minimum recommended for a thickener with nectar texture (1.2%) to a maximum recommended for a thickener with pudding texture (4.5%). The manufacturer’s recommendations are not very objective measurements (“ladles”), which imply a high degree of subjectivity. In addition, intermediate concentrations, as well as very high (9%) and a very low concentration (0.5%) were added in order to obtain a concentration/viscosity curve as accurate as possible. In this way, the viscosity of the 2 gum-based thickeners could be compared with the same concentration values. All the concentrations tested are shown in Table 2.

**Table 2:**
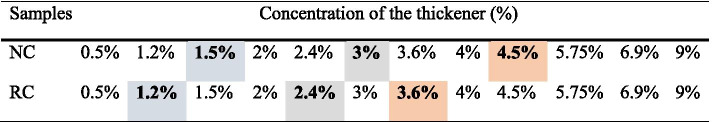
Concentrations of gum-based thickeners made with water

The manufacturer’s recommendations for obtaining the different textures are shown in blue for nectar, green for honey, the red for pudding

All the formulations were prepared in triplicate using 200 ml of water, and placed in a 200 ml beaker after preparation. Following preparation, the samples were left to rest for 10 min before their subsequent analysis.

### Rheological analysis

A stress-controlled rheometer (MCR 301, Anton Paar Physica, Austria) was employed using a CC17 coaxial cylinder geometry.

In order to assess the rheological behavior of the thickened liquids as a function of the shear rate, flow curves were drawn with a range between 0.01 and 200 s^− 1^, plotting the shear stress and viscosity against the shear rate. Viscosity was determined at a shear rate of 50 s^− 1^ obtained from each concentration’s flow curve. The temperature of the samples varied between 22 °C and 25 °C, trying to emulate the most common temperature conditions during home and clinical consumption [12]. Three specimens of each sample were measured for each determination. Rheological measurements were calculated after 10 min of sample preparation. We compared the average viscosity of two thickeners based on gum (NC vs RC) at 50 s^− 1^.

### Sensory analysis

Four triangular sensory tests were performed, evaluating the ability to recognize differences between nectar and honey texture, using only commercial gum-based thickeners (both RC and NC) with similar organoleptic qualities [13]. We discarded starch-based thickeners because they add color and flavor to the water, so panelists could differentiate them regardless of texture. The analyses were carried out individually with the participants of both panels (see below) seated. A tasting room was set up in the nursing home with four chairs and four tables separated by screens. Prior to the test, the methodology of the sensory test was explained. Two different types of panelists were selected: 1) Panel A: formed by 29 people under 65 years old (22 were women and 7 men), all of them health professionals from a nursing home. The average age of this panel was 36 years old. 2) Panel B: formed by 26 people over 65 years old institutionalized in same the residence (14 women and 12 men) with cognitive capacities in good condition and without pathologies that could affect the tasting result. The average age of this panel was 81 years old. First, the thickeners were compared using the concentrations recommended by the manufacturer for nectar texture (test 1) and honey texture (test 3). In addition, two other tests were performed at the same concentration with the two thickeners for nectar texture (test 2) and honey texture (test 4). Table 3 shows the concentration and viscosity of each of the samples in the 4 triangular sensory tests.

**Table 3:** Viscosity and concentration of thickeners compared in the triangular tests

Texture	Sample 1	Sample 2	Sample 3
Test 1 (Nectar)	233.9 (4) mPa·s (NC 1.5%)	131.7 (7) mPa·s (RC 1.2%)	131.7 (7) mPa·s (RC 1.2%)
Test 2 (Nectar)	233.9 (4) mPa·s (NC 1.5%)	183.9 (1) mPa·s (RC 1.5%)	183.9 (1) mPa·s (RC 1.5%)
Test 3 (Honey)	491.3 (1) mPa·s (NC 3%)	292.0 (3) mPa·s (RC 2.4%)	292.0 (3) mPa·s (RC 2.4%)
Test 4 (Honey)	491.3 (1) mPa·s (NC 3%)	356.6 (6) mPa·s (RC 3%)	356.6 (6) mPa·s (RC 3%)

Viscosity was presented in means (*SD* standard deviations) and concentration of thickeners in brackets grams of thickener per 100 ml of water

For each test, three 50 ml opaque chalices containing two similar RC samples and one different NC sample were presented on a plate, all of them coded. Each panelist had to single out the different sample. The samples were presented in white plastic cups in order to minimize possible differences in color between samples of different commercial thickeners. Both panels performed all the tests.

### Ethical aspects

The study was approved by the local ethics committee (Research Ethics Committee of the Autonomous Community of Aragon: CEICA) (registration number C.P.-C.I. PI15/0331). All participants signed an informed consent form before participating in the study. All methods were carried out in accordance with relevant guidelines and regulations.

### Statistical analysis

The statistical software IBM® SPSS® Statistics 25 was used. Quantitative variables were described by means (SD standard deviations), and qualitative variables by means of proportions. It was considered that no variable followed the normal distribution since the number of determinations was 3. Differences were considered significant with a *p* < 0.05.

Means were compared using the non-parametric Kruskal Wallis and Mann-Whitney U tests.

The sensory tests were evaluated according to the significance tables of the UNE-ISO 6658 standard [13]. The comparison between the proportions of participants who discriminated or not the textures in the sensory tests were made by means of Chi^2^ test.

## Results

### Viscosity curves of thickeners in water

**Fig. 1:**
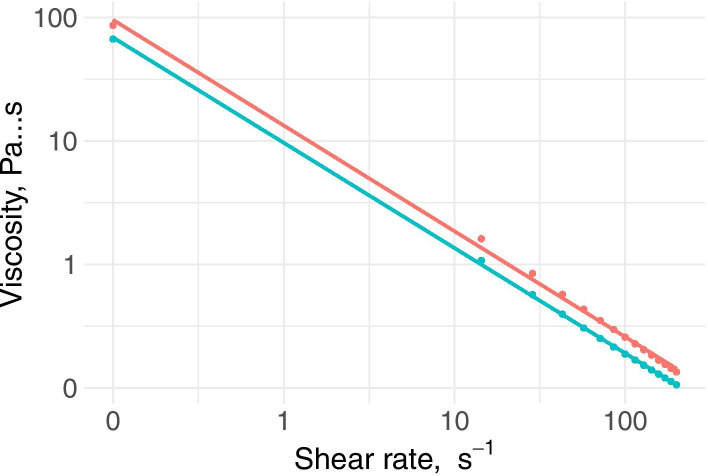
Graphical representation of the viscosity curve with the shear rate at a concentration of 3%

The results obtained when drawing the viscosity versus shear rate curves (in a logarithmic scale) with a concentration of 3% for the two gum-based thickeners are shown in Fig. 1. These curves show that the viscosity of the thickeners under study decreased with the shear rate, indicating non-Newtonian and pseudoplastic behavior. At low shear rates (0.0998 s^− 1^), the viscosity was 84.3 mPa·s for NC and 68.9 mPa·s for RC. When the shearing speed approached 20 s^− 1^, the viscosity dropped considerably: to 820 mPa·s for NC and 580 mPa·s for RC. At 50 s^− 1^, the viscosity dropped even further: to 491 mPa·s for NC and 356 mPa·s for RC. When the shearing speed reached the maximum of our measurement (200 s^− 1^) the viscosities of all were close to zero.

The average viscosity achieved by each type of thickener at a shear rate of 50 s^− 1^ with different concentrations is shown in Table 4. These data were obtained from the flow curves.

**Table 4:**
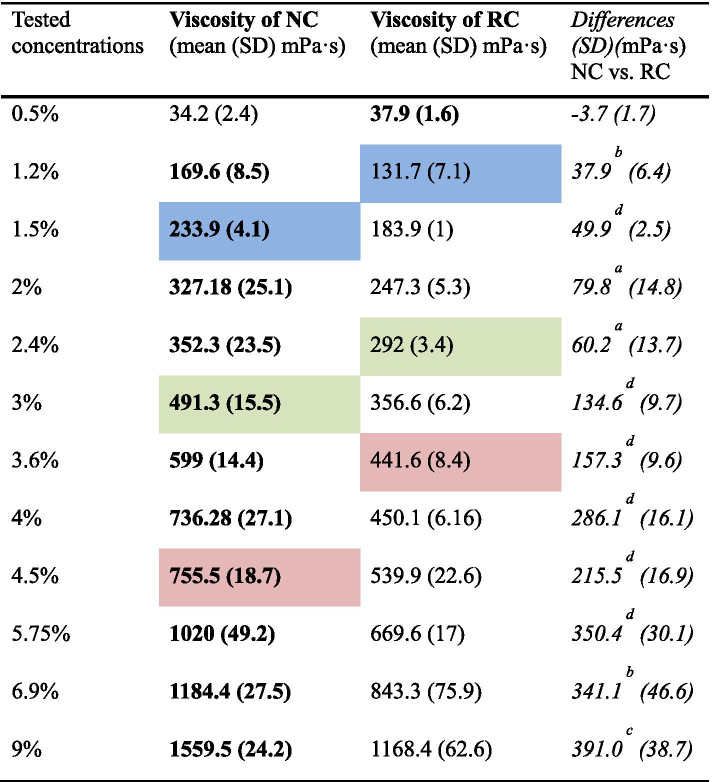
Viscosity comparison between gum-based thickeners at a 50 s^− 1^ shear rate in different concentrations

Viscosity was presented in mean (Standard Deviation)

The manufacturer’s recommendations for obtaining the different textures are shown in blue for nectar, green for honey, the red for pudding

In bold: Higher viscosity values obtained between the different concentrations of NC and RC thickeners

In italics: Average differences

Significance level: ^a^*p* < 0.05, ^b^*p* < 0.01, ^c^*p* < 0.001 and ^d^*p* < 0.0001

With the same concentration of thickener, each of them presented a different viscosity at 50 s^− 1^. NC reached a higher viscosity than RC in all concentrations (*p* < 0.05), except with the concentration of 0.5%. The dispersion values reflected in the standard deviation of the means obtained in the three repeated samples of the same concentration were higher with elevated concentrations.

### Sensory analysis

Table 3 shows the concentrations and viscosities of each sample evaluated by the different sensory analysis tests.

Sensory Test 1 compared two viscosities in the nectar texture range by analyzing one NC and two RC samples with the concentration recommended by the manufacturer for nectar texture (NC 1.5% with a mean (SD) viscosity of 233.9 (4) mPa·s and RC 1.2% with a viscosity of 131.7 (7) mPa·s). In this test, 26 out of 29 panel A tasters (young adults) detected the most viscous sample (89.6%) (*p* < 0.0001) and 22 out of 26 panel B tasters (elderly) detected the most viscous sample (84.6%) (*p* < 0.0001). Elderly and young participants discriminated among the samples in a very similar way (89.6% vs 84.6%, *p* = 0.69).

Sensory Test 2 again compared two viscosities in the nectar texture range but with the same concentration, one sample of 1.5% NC with a mean (SD) viscosity of 233.9 (4) mPa·s (nectar texture) and two samples of RC with the same concentration (1.5% with a viscosity of 183.9 (1) mPa·s). Of the 29 members of Panel A (young adults), 21 detected which sample was different (72.4%) (*p* < 0.0001). Fourteen of the 26 nursing home elderly participants detected the difference (53.8%) (*p* < 0.05). The proportion of patients who detected the difference tended to be significantly higher in young adults vs. elderly (72.4% vs. 53.8%; *p* = 0.17).

Sensory Test 3 compared two viscosities in the honey texture range with the concentration recommended by manufacturer; one sample of NC 3% with a viscosity of 491.3 (1) mPa·s and two samples of RC 2.4% with a viscosity of 292 (3) mPa·s. Of the 29 young adults in panel A, 27 detected which was the different sample (93.1%) (< 0.0001). Of the 26 older adults in Panel B, 19 detected the difference (73.1%) (*p* < 0.0001). The differences in discrimination between young and older adults were at the limit of statistical significance (93.1% vs. 73.1%, *p* = 0.069).

Finally, Sensory Test 4 compared two viscosities in the honey texture range but with the same concentration both thickeners; one sample of 3% NC with a viscosity of 491.3 (1) mPa·s (honey texture) and two samples of RC with the same concentration (3% with a viscosity of 356.6 (6) mPa·s). Of the 29 members of Panel A (healthy adult), 25 detected the different sample (86.2%) (*p* < 0.0001), while of the 26 elderly, 14 detected the difference (53.8%) (*p* < 0.05). The differences between groups in this case were statistically significant (86.2% vs 53.8%; *p* = 0.016).

**Fig. 2:**
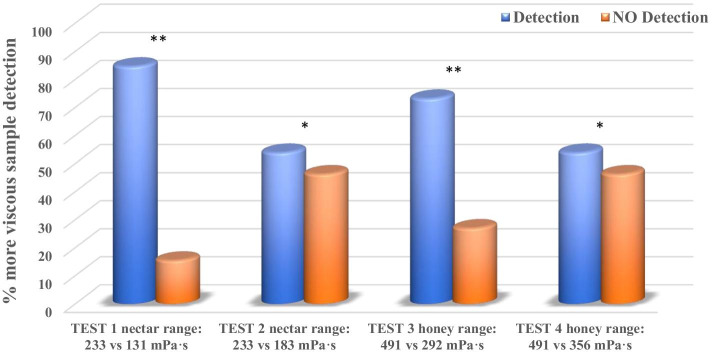
Comparison of percentage of correct detections of viscosity differences in institutionally elderly people. Significance level (*p < 0.05, **p < 0.0001)

**Fig. 3:**
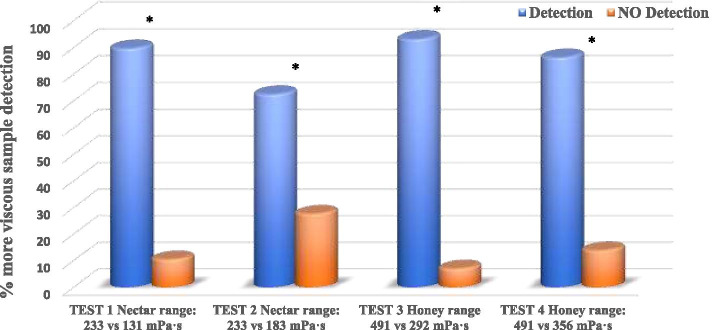
Comparison of percentage of correct detections of viscosity differences in young caregivers. Significance level (*p < 0.05, **p < 0.0001)

The percentage of institutionally elderly people who detected the most viscous sample within the nectar and honey texture ranges is presented in Fig. 2 while the Fig. 3 reflects the percentage of correct answers in the healthy adult population.

## Discussion

In this study, a sensory test was performed involving adults and institutionalized elderly people to evaluate whether an individual could discern small differences in viscosity in the same range of texture according to the National Dysphagia Diet Task Force [5] among different thickeners based on gums. The rheological properties of thickeners depend on their concentration and shear rate (swallowing strength). The understanding of these characteristics can facilitate an individualized prescription. The obtained results showed that the rheological behavior of the tested thickeners is non-Newtonian (pseudoplastic), meaning that the viscosity decreases when an external shear force is applied. In relation to the swallowing process, if the food bolus is a pseudoplastic fluid, its viscosity is altered by the propulsion of the tongue or by pharyngeal compression during swallowing, thus decreasing its viscosity [14]. This shear force exerted during swallowing on the alimentary bolus is not the same in a young adult as in an elderly person. Not all products used in clinical practice for the patient with dysphagia present this pseudoplastic behavior [15]. However, this pseudoplastic behavior has been described for the majority of thickened fluids [16].

Non-Newtonian rheological behaviors have also been described when analyzing the behavior of thickeners based on xanthan gum, maltodextrins, and gum arabic [9], as well as with thickeners based on gums, starch, or a mixture of both [17]. This rheological behavior of thickeners has clinical implications since the elasticity of the bolus contributes to a more pleasant and safer swallowing [18]. Gum-based thickeners have the highest viscosity at slow shear rates, so they may be safer for the elderly and for patients with motor dysphagia who exert less force during swallowing. This behavior its similar to describe by Seo et al. [7].

NC has a higher viscosity than RC at the same concentration level. When analyzing the thickeners viscosity dissolved in water at 50 s-1, NC reached a higher viscosity than RC with all concentrations except with the 0.5% one. This fact is surprising because the manufacturers for a nectar texture recommend less quantity for RC than for NC, and this is probably due to the fact that the accepted range of viscosity for nectar texture by NDD is very wide [19]. The same happens with the honey and pudding texture. Moreover, the higher the amount of grams of commercial thickener used, the greater the difference between NC and RC. These findings have clinical utility because they indicate that thickeners of different composition should not be interchanged because the handling is different. This practice can occur in places with a high incidence of dysphagia, and various thickeners are used such as nursing homes.

These results coincide with those of Park et al. [20] who observed that the viscosities obtained in two thickeners, one guar gum-based and the other one xanthan gum-based (both at 1% in water), were similar. However, as the concentration increased, the guar gum-based thickener reached a higher viscosity than the xanthan gum-based thickener. This could explain, in a way, why the NC thickener (based on xanthan gum, guar gum and maltodextrins) displayed, in our work, statistically significant higher viscosities with concentrations 1% and over than the xanthan gum and maltodextrin (RC) based thickener.

Sopade et al. [17] analyzed thickeners based on xanthan gum, whose viscosity results were lower in comparison with those of the present work (NC and RC). They also analyzed a thickener based on guar gum, which reached the highest viscosity values measured at 50 s^− 1^ with the lowest concentrations (2452.1 mPa·s with a concentration of 1.8%). In the same way, the study by Seo et al. [7] found that a thickener based on xanthan and guar gums reached higher viscosity than a thickener containing only xanthan gum at the same concentration. This suggests a higher thickening power of guar gum compared to xanthan gum.

In our study we observed that both thickeners could obtain water viscosities corresponding to nectar and honey textures (around 1000 mPa^.^s) but the pudding texture was reached with difficulty (> 1750 mPa^.^s). This limitation in its potency could be of relative clinical importance. According to Clavé and García [21], the textures most commonly used in prescriptions by doctors are nectar (60%) and honey (33%), with the pudding texture being the least prescribed (6%). This may be due to the fact that in very thick liquids (pudding textures) patients report poor tolerance and decrease in their liquid intake in addition to increasing their level of satiety [21, 22]. Moreover, a higher viscosity in the fluid will mean a greater shear effort in the swallowing process [23], and viscosities higher than 1000 mPa^.^s (and even 800 mPa^.^s) do not confer many benefits, because they no longer improve safety and efficiency in swallowing according to several recent articles [24, 25].

The combination of a rheological and sensorial analysis in institutionalized elderly offers for the first time a clear insight into the elderly capacity of detecting viscosities variations and warrants their application on clinical practice, beyond the classic texture classifications. Differences in rheological parameters with similar concentrations were detected by both panel A (young adults) and panel B (elderly) during the sensory study. When the differences in viscosity were high and therefore more distinct, they were perceived by almost all panelists (A and B). However, when they were minimal, it was much more difficult for the elderly in panel B to detect them (detection at the limit of statistical significance), although the great majority of non-elderly adults continued to perceive them. The result of the elderly participants can be explained by the fact that the difference in viscosity with these concentrations was less than the corresponding one according to the manufacturer and the shear force exerted during the swallowing process is less than that of an adult [26] or their ability to evaluate differences in viscosity is reduced.

Steele and Van Lieshout [27] suggested that motor and neurological ability decreases with advancing age, so changes in the ability to differentiate viscosities may appear, indicating that larger cohort studies are needed. Clinically this clearly means that the force exerted by the mouth is different in healthy adults (panel A) and in the elderly (panel B). The sensory analysis showed that classification of textures (nectar, honey and pudding) probably needs a review. Zhong et al. [11] carried out a similar study in university students, which can detect more than three viscosity increases in the nectar and honey ranges by NDD. They suggested that current viscosity ranges by NDD for categorizing the thickened levels might be too broad. In the same way, Steele et al. [10] also observed similar results in healthy individuals. The authors even proposed that people might be able to perceive at least four grades of increasing apparent viscosity within the nectar and honey-thick range by NDD. So, they suggested the creation of subcategories within the established ranges. We should take into account the real capacity of an individual to detect viscosities. It is verified with our study that the differences in viscosity, even when they are small, are detectable by institutionalized elderly people. Therefore, the NDD classification of nectar, honey and pudding probably should be refined. The viscosity ranges could be narrower to better suit the needs of each patient. Currently, the latest initiative of the IDDSI consists of the creation of four new ranges of liquids textures but uses qualitative methods with a syringe, instead of quantitative methods like rheological measurements [28]. However, this testing methods still needs more evidence before to be to recommend, as suggested by a recent review [29]. There are few studies that perform rheological tests of the IDDSI classification [28, 30, 31]. Those studies investigated the relationship between the viscosity measured by a rheometer and the amount of remaining sample in the syringe measured by IDDSI flow test for thickened water samples prepared with different food thickeners. Each work uses various thickeners with different compositions showing apparent viscosity at 50 s^− 1^ of approximately on average 60, 150 and 300 mPa^.^s for IDDSI levels 1, 2 and 3 respectively. With these results, it could be considered that the levels proposed by IDDSI would also show very wide ranges measured by rheometric. In our sensory study we have used the nectar (131–292 mPa^.^s) and honey (356–491 mPa^.^s) textures according to the NDD classification, which could correspond to levels 2 and 3 of the IDDSI classification according to these articles.

To our knowledge our study is the first one, in which a sensory test is performed in institutionalized elderly people and it is observed that they are able to distinguish small variations in viscosity despite physiological impairment due to age.

However, there are some limitations in our study. The variations in the sensory tests may not be entirely associated with rheological differences since it cannot be ruled out that the taste, smell or other sensory characteristics may change slightly with different concentrations. Moreover, in sensory tests, the population studied did not have swallowing disorders; therefore, the results cannot be directly extrapolated to patients with different types of dysphagia, although there is no reason to think that the results could be different. On the other hand, it was not possible to determine whether the different rheological characteristics was associated with greater safety during swallowing. Studies using videofluoroscopy would be necessary to evaluate the risk of aspiration with the different viscosities.

## Conclusions

We have observed that both institutionalized elderly and their younger caregivers are able to discern small differences in viscosity in nectar and honey textures. These results are important on a clinical level because it could signify that the classification of nectar, honey and pudding should be revised through rheological analyses with less wide ranges. The sample of healthy adults detected small differences in viscosity more effectively, so the force exerted by the mouth during swallowing may be different between healthy adults and the elderly. Furthermore, at the same concentration, each thickener produces a different viscosity, detectable even by institutionalized elderly people. Thickeners are not interchangeable with each other even if they belong to the same group (gum-based thickeners). Each one has a different composition, which influences its power to increase the viscosity of the water, and requires a different handling.

Further studies using video fluoroscopy or other clinical methods are needed to verify whether the rheological characteristics of thickeners influence the safety, adherence, or patient preference for different thickeners according to the type or intensity of their dysphagia.

## Data Availability

The datasets analyzed in the current study will be available from the corresponding author on reasonable request, through a research agreement that includes the approval of the Ethics Committee of Aragón for Clinical Research (CEIC-A).

## Abbreviations

NC: Nutilis Clear®
RC: Resource Clear®
NDD: National Dysphagia Diet Task Force
mPa·s: milliPascals per second
CEICA: Research Ethics Committee of the Autonomous Community of Aragon
SD: Standard Deviation
IDDSI: International Dysphagia Diet Standardization Initiative
